# Prognostic factors and a preliminary prognostic model in anti-GAD antibody-associated epilepsy

**DOI:** 10.3389/fimmu.2026.1738062

**Published:** 2026-02-04

**Authors:** Lin Bai, Nan Lin, Xiaochuan Zhang, Haitao Ren, Le Zhang, Jie Lu, Huiqin Liu, Yun Cai, Yueli Zou, Siyuan Fan, Qiang Lu, Hongzhi Guan

**Affiliations:** 1 Department of Neurology, Peking Union Medical College Hospital, Peking Union Medical College and Chinese Academy of Medical Sciences, Beijing, China; 2 Beijing Huiren Technology Development Co., Ltd., Beijing, China; 3 Department of Neurology, Nanjing Brain Hospital, The Affiliated Brain Hospital of Nanjing Medical University, Nanjing, Jiangsu, China; 4 Department of Neurology, Henan Provincial People’s Hospital, Zhengzhou, Henan, China; 5 Department of Neurology, Affiliated Hospital of Hebei University, Baoding, Hebei, China; 6 Department of Neurology, The Second Hospital of Hebei Medical University, Shijiazhuang, Hebei, China

**Keywords:** autoimmune, epilepsy, glutamic acid decarboxylase, prognostic model, temporal lobe epilepsy

## Abstract

**Background:**

Prognostic determinants in anti-glutamic acid decarboxylase (GAD) antibody-associated epilepsy remain unclear, and no validated predictive model exists. We aimed to identify prognostic factors and develop a predictive model.

**Methods:**

This multicenter cohort included patients diagnosed with anti-GAD antibody-associated epilepsy before September 2024. Data encompassed demographics, seizure semiology, cellular and serological parameters, neuroimaging and electrophysiological findings, and treatment regimens. Favorable outcome was defined as seizure-free for ≥12 months following immunotherapy and antiseizure medications, poor outcome was defined as persistent seizures. Prognostic factors were analyzed and a predictive model was constructed.

**Results:**

Among 91 patients, 22 (24%) achieved seizure freedom, whereas 69 (76%) continued to experience seizures despite appropriate treatment. Poor prognosis was associated with focal seizures (50% vs. 81%, *p* = 0.004), temporal lobe epilepsy (TLE) (23% vs. 75%, *p* < 0.001), musicogenic epilepsy (n = 5, all with poor seizure control), and higher seizure frequency [≥1 seizure/month (67% vs. 97%, *p* < 0.001)]. In contrast, a shorter disease duration from symptom onset to diagnosis [3 (IQR 0.9–26.0) vs. 8 (IQR 1.5–36.0) months, *p* = 0.025], a shorter interval to initiation of immunotherapy [3 (IQR 1.0–14.0) vs. 7 (IQR 1.9–27.3) months, *p* = 0.005], higher CD8^+^T-cell counts (829.5 ± 473.9 vs. 619.5 ± 338.6 cells/µL, *p* = 0.035) were associated with favorable outcomes. Multivariate logistic regression identified TLE (OR = 0.098, 95% CI: 0.028–0.341, *p* < 0.001) and seizure frequency (OR = 0.067, 95% CI: 0.010–0.450, *p* = 0.005) as independent predictors of prognosis. The prognostic model based on these two variables demonstrated good discrimination (AUC = 0.807, 95% CI: 0.696–0.919, *p* < 0.001) and calibration (Hosmer–Lemeshow *χ²* = 0.124, *p* = 0.740), with sensitivity of 81.8%, specificity of 72.5%, and overall accuracy of 74.7%. Internal validation with bootstrapping confirmed model stability. Risk stratification further classified patients into low- (8.7%), intermediate- (49.3–58.9%), and high-risk (93.6%) groups for poor prognosis.

**Conclusion:**

Focal seizures, TLE, and higher seizure frequency were associated with poor prognosis, whereas early diagnosis, timely treatment, and higher peripheral CD8^+^T-cell counts were associated with favorable outcomes. TLE and seizure frequency independently predicted clinical outcomes in anti-GAD antibody-associated epilepsy. The logistic regression model effectively stratified patients, identifying those likely to achieve seizure freedom versus refractoriness.

## 1 Introduction

Anti-glutamic acid decarboxylase (GAD) antibody-associated neuroimmune disorders (GAD-ANDs) are autoimmune neurological conditions characterized by the presence of anti-GAD antibodies and typical clinical syndromes, including limbic encephalitis (LE), epilepsy (EP), stiff-person syndrome (SPS), autoimmune cerebellar ataxia (ACA), or overlapping phenotypes (1). Among these, GAD-EP and GAD-LE account for 52.9%-61.8% of GAD-ANDs, constituting the predominant clinical phenotypes (2, 3).

The association between anti-GAD antibodies and epilepsy was first reported in 1988 (4), with subsequent studies confirming their presence in patients with drug-resistant epilepsy by 1998 (5). Anti-GAD antibodies are present in 2.58% of all epilepsy patients (6), with higher prevalence in cryptogenic focal epilepsy (7.7%) (6), adult-onset temporal lobe epilepsy (TLE) (8.7%) (7), and refractory focal epilepsy (15.7%) (8). In 2020, the International League Against Epilepsy (ILAE) recognized GAD-EP as autoimmune-associated epilepsy, distinct from acute symptomatic seizures secondary to autoimmune encephalitis (9). Notably, patients with GAD-LE who experience seizures often progress to chronic drug-resistant epilepsy. Longitudinal studies suggest similar seizure outcomes between LE patients with seizures and isolated GAD-EP (3), supporting their combined analysis as a single clinical phenotype in prognostic modeling.

GAD-EP involves complex autoimmune mechanisms, including autoantibodies, B cells (10, 11), and T cells (12, 13). Cellular and serological parameters, such as lymphocyte counts and immunoglobulin levels, have been increasingly applied in neuroimmune disorders to guide individualized therapy. However, the relationship between peripheral immune cell counts or immunoglobulin levels and the prognosis of GAD-EP remains unclear.

Although 55.6%–90% of patients initially respond to immunotherapy and antiseizure medications (ASMs) (≥50% seizure frequency reduction), sustained seizure freedom is achieved in 0–36.7% (2, 3, 14–19), indicating that controlling seizures in these patients remains a major clinical challenge. Acute or subacute onset and early immunotherapy may improve outcomes, whereas focal or high-frequency seizures predict poor prognosis (19). While predictive models have been proposed for autoimmune encephalitis or epilepsy mediated by neuronal surface antibodies (20, 21), no such model currently exists for GAD-EP.

To address these gaps, we comprehensively collected demographic, clinical, immunological, neuroimaging, and electrophysiological data from patients with GAD-EP to identify prognostic factors and develop a logistic regression–based predictive model. The model supports risk-based patient stratification to inform individualized therapy.

## 2 Methods

### 2.1 Study population

**Figure 1:**
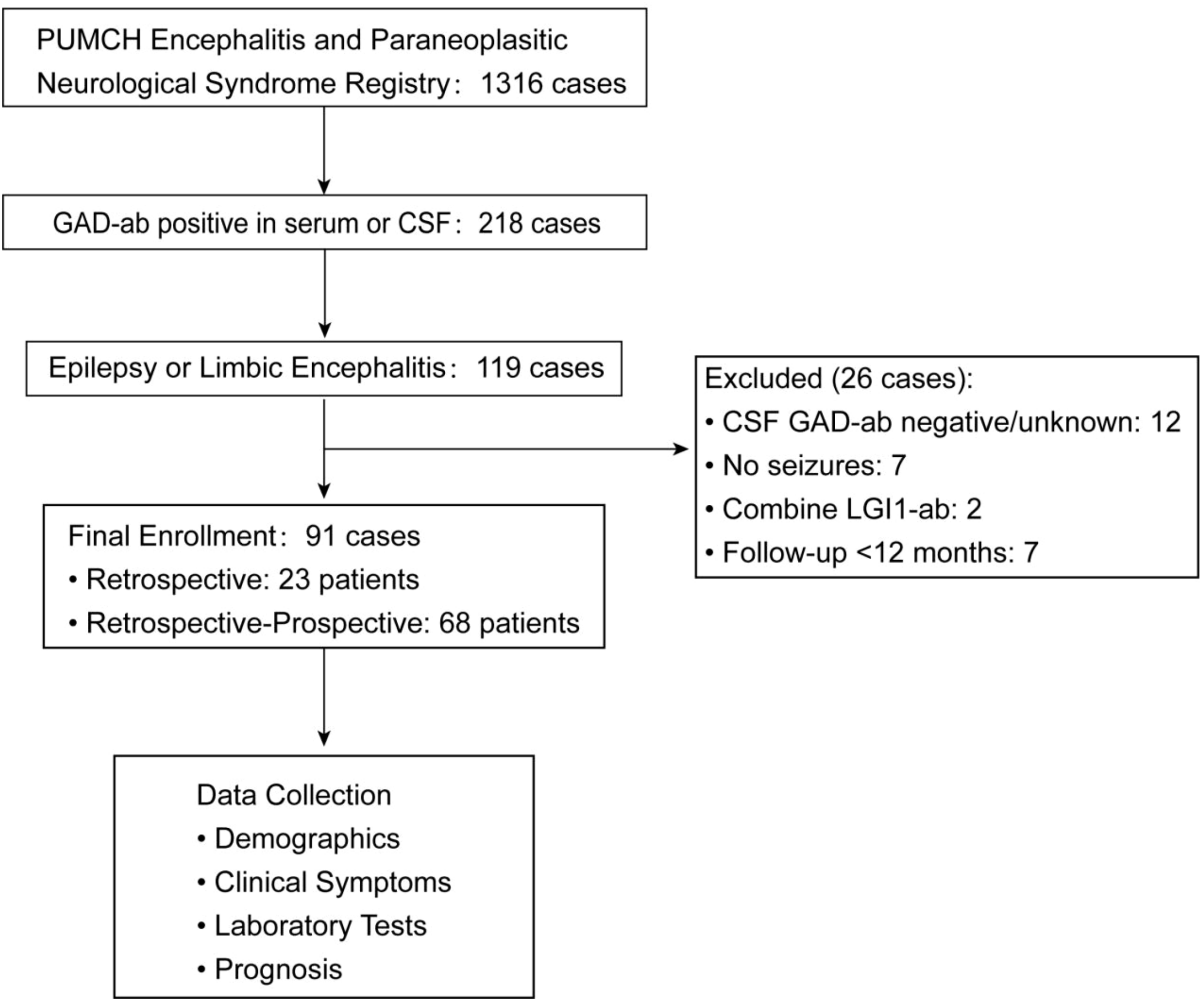
Patient enrollment flowchart. PUMCH, Peking Union Medical College Hospital; GAD-ab, anti- glutamic acid decarboxylase antibody; LGI1-ab, anti-leucine rich glioma inactivated 1 antibody; CSF, cerebrospinal fluid.

This multicenter retrospective-prospective cohort study included patients with GAD-EP and GAD-LE. A total of 1,316 patients were enrolled before September 2024, of whom 218 tested positive for anti-GAD65 antibodies in serum or cerebrospinal fluid (CSF). Among these, 119 patients presented with epilepsy or limbic encephalitis. Patients were excluded for unavailable or negative CSF anti-GAD65 antibody results (n = 12), limbic encephalitis without seizures (n = 7), coexisting anti-LGI1 antibodies (n = 2), or follow-up <12 months (n = 7). The final cohort comprised 91 patients (**Figure 1**).

### 2.2 Diagnostic criteria and outcome definition

Epilepsy was diagnosed according to the 2025 ILAE criteria (22). TLE was defined by typical temporal semiology with supportive EEG or MRI findings. LE was diagnosed based on the 2016 criteria proposed by Graus et al (23). A favorable outcome was defined as seizure freedom after immunotherapy and ASMs, whereas a poor outcome was defined as persistent seizures despite treatment. The minimum follow-up was 12 months, with a median of 24 months (IQR 14.5, 40).

### 2.3 Antibody detection

Anti-GAD65 antibodies were detected using a standardized commercial cell-based assay (CBA) (Euroimmun AG, Lübeck, Germany). Plasma or CSF (30μL) was incubated with biological substrates for 30 minutes at room temperature, followed by incubation with fluorescein isothiocyanate (FITC)-labeled anti-IgG and evaluation under fluorescence microscopy. All centers applied identical testing protocols, with confirmatory testing performed centrally when required.

### 2.4 Data collection

Collected variables included age at onset, gender, disease duration (from symptom onset to diagnosis), seizure semiology, comorbidities, neuroimaging findings, electroencephalography results, peripheral lymphocyte subsets, immunoglobulin levels, CSF parameters, time to immunotherapy initiation (from symptom onset), immunotherapy regimens, ASMs, and follow-up duration. Multicenter data were centrally registered and independently reviewed by two professional neurologists to ensure consistency across centers.

### 2.5 Statistical analysis

Variables with ≥20% missing data were excluded. Missing seizure frequency values (3 cases) were imputed using median values. Continuous variables were assessed for normality and summarized as mean ± standard deviation or median (interquartile range). Group comparisons used t tests or Mann-Whitney U tests for continuous variables and chi-square or Fisher’s exact tests for categorical variables. Multivariable logistic regression was used to identify independent prognostic factors and construct the predictive model. Model discrimination was assessed using receiver operating characteristic (ROC) curves, and internal validation was performed with 1,000 bootstrap resamples. Analyses were conducted using SPSS 21.0 and Python 3.11.

## 3 Results

### 3.1 Cohort characteristics

The cohort included 91 patients (female-to-male ratio, 3.8:1), with a mean age at onset of 33.8 ± 14.9 years and a median disease duration of 8 months (IQR 1.5–36). TLE was observed in 63% of patients, and musicogenic epilepsy in 6%. Seizure frequency was daily in 49%, weekly in 24%, and monthly in 17% of patients.

Autoimmune comorbidities were present in 50% of patients, most commonly autoimmune thyroid disease and type 1 diabetes mellitus (T1DM), with less frequent conditions including vitiligo, myasthenia gravis, and autoimmune anemia. Psychiatric comorbidities (e.g., anxiety state and bipolar disorder) occurred in 23%. Neoplasms were identified in six patients, including thymoma (n=2), teratoma (n=2), breast cancer (n=1), and gastric neuroendocrine tumor (n=1).

Median serum and CSF anti-GAD65 antibody titers were 1:100. Peripheral lymphocyte subsets and serum immunoglobulin levels were generally within normal reference ranges. CSF pleocytosis was present in 19% patients, and brain MRI abnormalities occurred in 58% of patients, including temporal lobe atrophy or hypometabolism in 26%.

Eighty-seven patients received immunotherapy. Treatment was initiated immediately after diagnosis in 75 patients (86%), delayed by 1–4 months in 8 (9%), and administered empirically before diagnostic confirmation in 4 (5%). First-line therapy consisted of corticosteroids and/or intravenous immunoglobulin (IVIG), administered as monotherapy or in combination. Twenty-three patients with inadequate response to first-line therapy were treated with B-cell-depleting agents. Two patients underwent epilepsy surgery, neither of whom achieved seizure freedom (**Table 1**).

**Table 1:** Clinical characteristics and prognostic factors.

Variable	Total	Seizure-free	Seizure	*p*
Gender				1.000
Female	72 (79.1)	18 (81.8)	54 (78.3)	
Male	19 (20.9)	4 (18.2)	15 (21.7)	
Age at onset (y)	33.80 ± 14.90	38.77 ± 16.31	32.22 ± 14.18	0.072
Disease duration (onset to diagnosis, m)	8 (IQR 1.5–36)	3 (IQR 0.88–26)	12 (IQR 2–48)	0.025*
Clinical phenotype				0.451
EP/LE	73 (80.2)	17 (77.3)	56 (81.2)	
+ SPS	6 (6.6)	3 (13.6)	3 (4.3)	
+ ACA	7 (7.7)	1 (4.5)	6 (8.7)	
+ SPS+ACA	5 (5.5)	1 (4.5)	4 (5.8)	
Seizure types				
GTCS	66 (72.5)	16 (72.7)	50 (72.5)	0.981
Focal seizures	67 (73.6)	11 (50)	56 (81.2)	0.004*
TLE	57 (62.6)	5 (22.7)	52 (75.4)	0.000*
Musicogenic epilepsy	5 (5.5)	0	5 (7.2)	0.331
Seizure frequency				0.000*
<1 per month	9 (10.2)	7 (33.3)	2 (3.0)	
At least monthly seizures	79 (89.8)	14 (66.7)	65 (97.0)	
Psychiatric disorders	21 (23.1)	5 (23.8)	16 (76.2)	1.000
Combined autoimmune diseases	45 (49.5)	11 (50)	34 (49.3)	0.953
T1DM	24 (26.4)	6 (27.3)	18 (26.1)	0.912
Autoimmune thyroid disease	24 (26.4)	7 (31.8)	17 (24.6)	0.506
Others	9 (9.9)	3 (13.6)	6 (8.7)	0.682
Neoplasms	6 (6.6)	2 (9.1)	4 (5.8)	0.629
CSF WBC				0.156
>5×10^6^/L	12 (18.8)	5 (31.3)	7 (14.6)	
≤5×10^6^/L	52 (81.3)	11(78.6)	41 (85.4)	
Initial peripheral lymphocyte subsets (normal range)				
CD3^+^T (940–2140 cells/µL),	1672.00 ± 743.16	1961.05 ± 916.36	1572.33 ± 653.26	0.092
CD4^+^T (550–1200 cells/µL)	873.34 ± 834.81	998.55 ± 440.34	830.76 ± 358.29	0.092
CD8^+^T (380–790 cells /µL)	673.33 ± 385.71	829.45 ± 473.86	619.50 ± 338.57	0.035*
CD19 (160–350 cells/µL)	297.06 ± 234.31	1.00 ± 0.45	283.20 ± 216.45	0.436
Initial serum immunoglobulin levels (normal range)				
IgM (0.4–2.3g/L)	0.97 ± 0.58	11.54 ± 4.78	0.96 ± 0.62	0.818
IgG (7–17g/L)	11.48 ± 4.97		11.47 ± 5.08	0.956
EEG				0.359
Abnormal discharge	68 (81.0)	14 (73.7)	54 (83.1)	
Slow wave	10 (11.9)	4 (21.1)	6 (9.2)	
Normal	6 (7.1)	1 (5.3)	5 (7.7)	
MRI and FDG-PET/CT				0.938
Limbic system involvement only	44 (48.4)	10 (45.5)	34 (49.3)	
Other brain regions involved	9 (9.9)	2 (9.1)	7 (10.1)	
Normal	38 (41.8)	10 (45.5)	28 (40.6)	
Immunotherapy	87 (95.6)	21 (95.5)	66 (95.7)	1.000
No immunotherapy	4 (4.4)	1 (4.5)	3 (4.3)	
Immunotherapy regimen				0.732
First-line immunotherapy	12 (13.8)	3 (14.3)	9 (13.6)	
First-line + immunosuppressants	50 (57.5)	13 (61.9)	37 (56.1)	
Immunosuppressants only	2 (2.3)	0	2 (3.0)	
Second-line immunotherapy	23 (26.4)	4 (19.0)	19 (28.8)	
Onset to immunotherapy initiation (m)	7(IQR 1.75–30)	3(IQR 1–14)	7(IQR 1.9–27.3)	0.005*
ASM types				0.088
≤2 ASMs	73 (83.9)	21 (95.5)	52 (80)	
>2 ASMs	14 (16.1)	1 (4.5)	13 (20)	
Follow-up time	30.57 ± 19.78	26.73 ± 19.78	31.8 ± 19.77	0.302

EP, epilepsy; LE, limbic encephalitis with seizures at onset; SPS, stiff-person syndrome; ACA, autoimmune cerebellar ataxia; GTCS, generalized tonic-clonic seizure; TLE, temporal lobe epilepsy; T1DM, type 1 diabetes mellitus; ASM, anti-seizure medication; m,months; y,years; *p < 0.05.

### 3.2 Prognostic factors

At a mean follow-up of 30.6 ± 19.8 months, 22 patients (24%) achieved seizure freedom. Univariate analysis demonstrated that focal seizures (*p* = 0.004), TLE (*p* < 0.001), and higher seizure frequency (≥1 seizure/month vs. <1 seizure/month; *p* < 0.001) were significantly associated with poor outcomes. All cases of musicogenic epilepsy were refractory, although this association did not reach statistical significance.

Favorable outcomes were associated with a shorter disease duration [3 (IQR 0.9–26.0) vs. 8 (IQR 1.5–36.0) months, *p* = 0.025], earlier initiation of immunotherapy [3 (IQR 1.0–14.0) vs. 7 months (IQR 1.9–27.3)months, *p* = 0.005], and higher CD8^+^ T-cell counts (829.5 ± 473.9 vs. 619.5 ± 338.6 cells/µL, *p* = 0.035) (**Supplementary Figure S1**).

Serum and CSF anti-GAD65 antibody titers were not associated with prognosis or the presence of TLE (**Supplementary Figure S2**). Patients with hippocampal atrophy or hypometabolism had a lower rate of seizure freedom than those without these abnormalities (17% vs. 27%), however, the difference was not statistically significant (*p* = 0.411). Combination therapy with corticosteroids plus IVIG was not significantly associated with improved prognosis compared with monotherapy (*p* = 0.133; **Supplementary Table S1**). Rituximab treatment was also not significantly associated with seizure-freedom (*p* = 0.571; **Supplementary Table S2**).

Variables significant in univariate analysis were entered into a multivariable logistic regression model. TLE (*p* = 0.001) and higher seizure frequency (*p* = 0.010) emerged as independent predictors of seizure freedom (**Table 2**).

**Table 2:** Multivariable logistic regression analysis of prognostic factors.

Variable	B	SE	p value	OR	95% CI
Disease duration (m)	-0.017	0.017	0.323	0.983	0.951-1.017
Focal seizurs	-1.046	0.943	0.267	0.351	0.055-2.231
TLE	-3.068	0.960	0.001*	0.046	0.007-0.035
Seizure frequency	-3.208	1.239	0.010*	0.040	0.004-0.459
CD8^+^T cell	0.001	0.001	0.114	1.001	1.000-1.003

TLE, temporal lobe epilepsy; m, month;seizure frequency was categorized as <1 seizure/month vs. ≥1 seizure/month; *p < 0.05; endpoint: seizure freedom (good prognosis).

### 3.3 Development of the prognostic model

**Figure 2:**
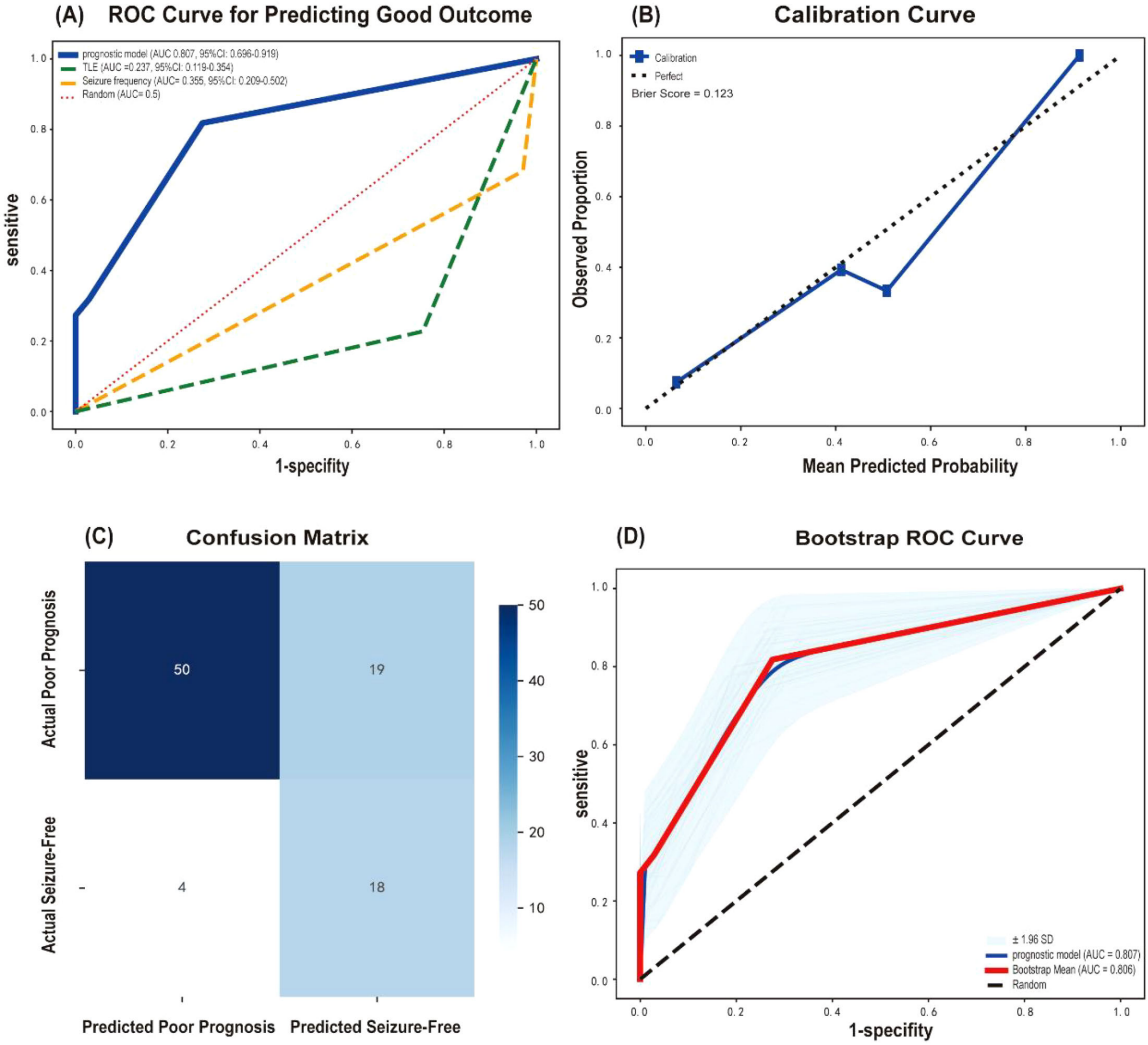
Performance evaluation of the prognostic model. Predictive outcome: seizure freedom **(A)** ROC curves of the model and individual predictors **(B)** Calibration curve **(C)** Confusion matrix **(D)** Bootstrap internal validation with 95% confidence intervals.

TLE and seizure frequency were included in a binary logistic regression model to predict seizure-free outcomes. Both TLE (OR = 0.098, 95% CI: 0.028–0.341, *p* < 0.001) and frequent seizures (OR = 0.067, 95% CI: 0.010–0.450, *p* = 0.005) were associated with lower likelihood of seizure-free. The predictive model was: *Logit (seizure-free probability) = 2.349-2.319×TLE-2.707×seizure frequency*. The model demonstrated good predictive performance with an AUC of 0.807 (95% CI: 0.696–0.919, *p* < 0.001). At the optimal cutoff value of 0.238, the sensitivity and specificity were 81.8% and 72.5%, respectively, with a positive predictive value (PPV) of 48.6%, a negative predictive value (NPV) of 92.6%, and an overall accuracy of 74.7%. The Hosmer-Lemeshow test indicated good calibration (*χ²* = 0.124, *p* = 0.724), and the Brier score was 0.123. Internal validation using 1,000 bootstrap resamples confirmed the stability of the model (**Figure 2**).

### 3.4 Development of a risk scoring system

A risk-scoring system was developed using two independent predictors: TLE and seizure frequency ≥1/month (1 point each; total 0–2). Predicted poor outcome probabilities were 8.7%, 49.3–58.9%, and 93.6% for low-, intermediate-, and high-risk groups, respectively (**Table 3**). High-risk patients (TLE with frequent seizures) had only 6.4% seizure-freedom rate (95% CI: 0.4–14.1%), indicating standard therapy is largely ineffective and novel strategies are needed. Low-risk patients (non-TLE with infrequent seizures) achieved 91.3% seizure freedom, suggesting standard therapy suffices.

**Table 3:** Risk stratification based on the prognostic model.

Feature			Assigned value
TLE			1
Non-TLE			0
seizure frequency ≥ once per month			1
seizure frequency < once per month			0
Epilepsy risk Group	Score	Predicted poor outcome	Observed poor outcome in cohort
Low risk	0	8.7%	0(0/6)
Intermediate risk	1	49.3-58.9%	61.2%(19/31)
High risk	2	93.6%	92.7%(51/55)

TLE, temporal lobe epilepsy.

## 4 Discussion

This study included 91 patients with GAD-EP and aimed to identify prognostic factors and develop a predictive model. We found that focal seizures, TLE, and high seizure frequency were associated with poor outcomes, whereas a shorter disease duration, earlier initiation of immunotherapy, and higher CD8^+^T-cell levels were associated with seizure-freedom. TLE and seizure frequency emerged as independent predictors of poor prognosis. A prognostic model including these two variables showed good predictive performance. Based on this model, a risk scoring system was developed and demonstrated high accuracy in distinguishing low- and high-risk patients. Clinically, the score may aid early risk stratification, enabling timely use of standard immunotherapy in low-risk patients while prompting consideration of alternative or novel treatment strategies in high-risk patients rather than repeated application of ineffective regimens.

TLE is the most common subtype of GAD-EP, typically associated with poorer outcomes compared with non-GAD-related mesial TLE (14). Higher seizure frequency or number of seizures before remission are established predictors of poor seizure control, drug resistance, and recurrence in epilepsy patients (24–26). We developed a predictive model incorporating these factors, which demonstrated high sensitivity and excellent negative predictive value and accurately identifies patients likely to achieve seizure freedom while minimizing misclassification of those with poor outcomes.

In our cohort, 9 patients (10%) met the criteria for LE and 82 (90%) had GAD-EP, with no difference in seizure outcomes (*p* = 1.000). CSF pleocytosis tended to be associated with better outcomes, although this did not reach statistical significance (42% vs. 21%, *p* = 0.139). Including LE and CSF-inflammatory patients in the prognostic model maintained high accuracy (88.9% and 75.0%), supporting the combination of LE and EP for modeling. Elevated CSF WBC persisted up to 36 months, and disease duration ≤ 36 months was associated with better prognosis (*p* = 0.035), emphasizing that early diagnosis and treatment during the inflammatory phase—whether presenting as acute LE or chronic epilepsy—improves outcomes. We propose that patients with LE previously underwent earlier antibody testing and treatment, but growing recognition of GAD-EP now enables similarly timely diagnosis and therapy, reducing prognostic differences between the two presentations.

We found that higher peripheral CD8^+^T-cell counts at initial presentation were associated with favorable prognosis, suggesting they may reflect disease immune activity. Higher counts may indicate an active phase, whereas lower levels could reflect the chronic stage. Patients with disease duration ≤36 months had higher counts than those with longer disease (702.2 ± 404.5 vs. 529.3 ± 234.6 cells/µL), although the difference was not statistically significant (*p* = 0.141). These findings support the potential of CD8^+^T-cell counts as a biomarker, warranting larger studies with longitudinal monitoring to validate and define an optimal cutoff.

Brain MRI showed limited prognostic value in our cohort. Although patients without hippocampal atrophy or hypometabolism had higher rates of seizure freedom, the difference was not statistically significant. Several factors may explain this: (1) limited sample size, reducing statistical power; (2) heterogeneous timing of imaging, as patients were scanned at different disease stages, introducing variability and highlighting the need for standardized assessments at fixed time points in future studies; (3) limited sensitivity of conventional MRI, since subtle hippocampal or functional damage in GAD-EP may precede visible atrophy. Previous studies have shown more extensive hypometabolism on FDG-PET in GAD-TLE compared with hippocampal sclerosis (27), suggesting that FDG-PET may offer greater sensitivity; (4) GAD-EP involves a complex interplay between structural injury and immune-inflammatory processes, with brain inflammation potentially driving seizure persistence beyond structural damage, although noninvasive current methods to assess tissue inflammation remain limited (28).

Management of GAD-EP remains challenging, with conflicting immunotherapy evidence: some studies report benefits from early corticosteroids or IVIG (19, 29), while others show limited effects (15, 30). In our study, different immunotherapy regimens showed no significant differences in seizure freedom. To address indication bias—where severe cases receive intensive treatment—we applied inverse probability of treatment weighting (IPTW) with logistic regression, adjusting for age, disease duration, clinical phenotype, TLE, seizure frequency, imaging atrophy, CD8^+^T-cell counts, and ASMs number. After adjustment, combination first-line therapy showed no superiority over monotherapy (OR = 0.42, 95% CI: 0.18–0.93, *p* < 0.05), and rituximab for inadequate first-line response showed no benefit (OR = 0.70, 95% CI: 0.33–1.45, *p* > 0.05). These findings warrant cautious interpretation given potential unmeasured confounders (economic factors, HLA background,etc.). Symptomatic treatments like cenobamate-clobazam show modest benefit (31), while vagus nerve stimulation and epilepsy surgery show limited efficacy (32). Randomized controlled trials are needed for definitive immunotherapy comparisons. Novel therapeutic approaches are urgently required.

This study has several limitations. First, the retrospective design may introduce information bias. Second, despite representing a relatively large East Asian GAD-EP cohort, only 22 patients (24.2%) achieved seizure freedom, limiting statistical power. Following the 10 events-per-variable principle, we included two predictors, constraining model complexity. Consequently, the model generates four discrete probability values with limited discrimination for intermediate-risk patients. Third, although bootstrap validation addresses optimism bias, external validation is lacking. Ongoing patient enrollment may identify additional predictors to improve risk stratification. Currently, this model serves as a preliminary screening tool requiring validation in independent cohorts.

In conclusion, we analyzed patients with GAD-EP from multiple centers and evaluated multidimensional predictive variables. Focal seizures, TLE, and high seizure frequency were associated with poor outcomes, while early diagnosis and treatment and higher CD8^+^T-cell counts predicted favorable prognosis. TLE and seizure frequency were identified as independent predictors, forming a prognostic model with good discriminative performance. The resulting risk-scoring system effectively distinguishes high- and low-risk patients, providing a practical tool for clinical risk stratification and treatment decision-making in GAD-EP.

## Data Availability

The raw data supporting the conclusions of this article will be made available by the authors, without undue reservation.
