# Supplementary material for: Prognostic factors and a preliminary prognostic model in anti-GAD antibody-associated epilepsy
Source: Front Immunol. 2026 Feb 4;17:1738062. doi: 10.3389/fimmu.2026.1738062 (PMC12913182; doi:10.3389/fimmu.2026.1738062)
Supplement: Supplementary file 1 [file Image1.pdf]

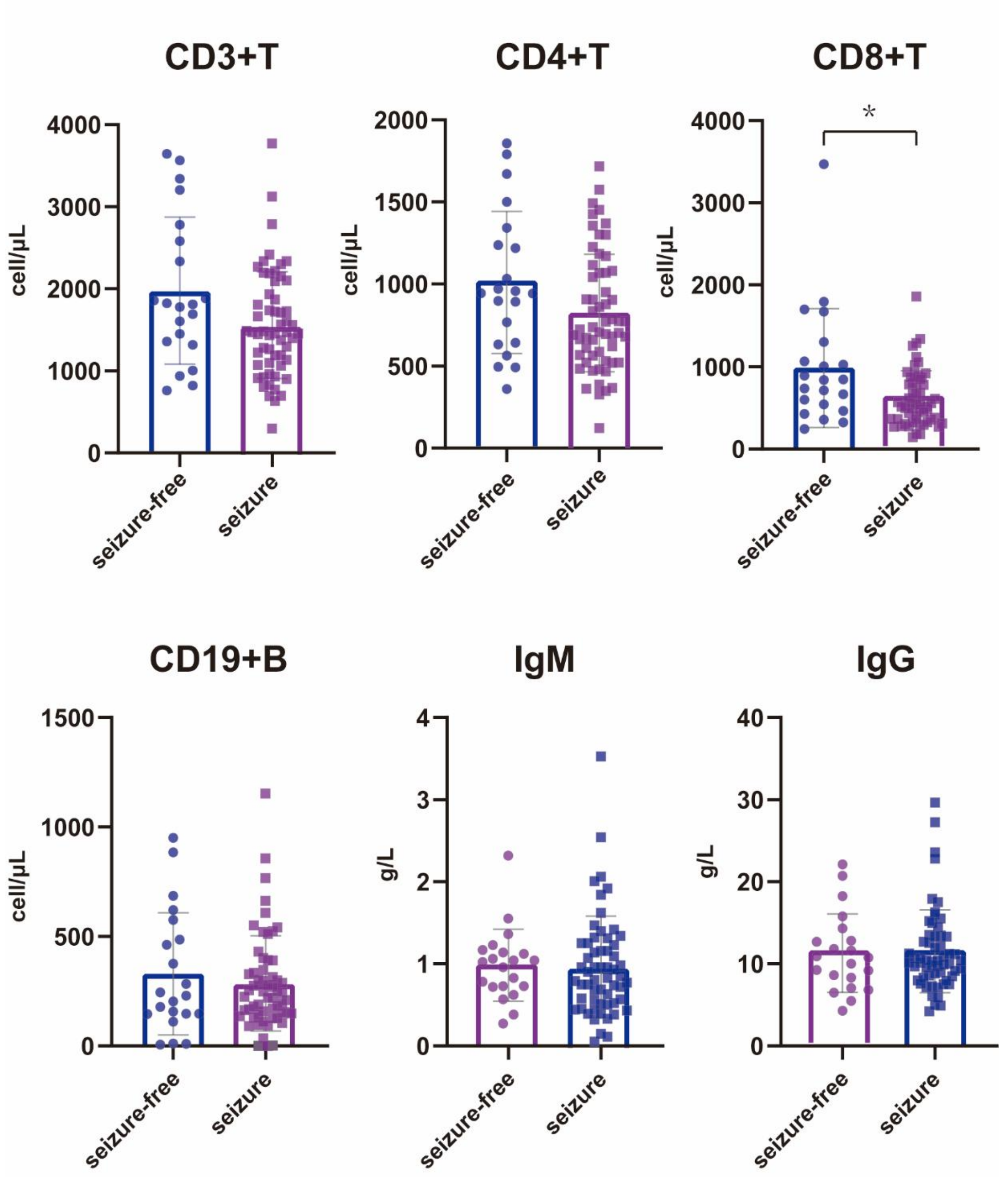

Figure S1. Peripheral blood T and B lymphocyte subsets and serum immunoglobulin levels at initial presentation.

Comparison of peripheral blood lymphocyte subsets ($CD3^{+}$ T, $CD4^{+}$ T, $CD8^{+}$ T, $CD19^{+}$ B cells) and serum immunoglobulins (IgM, IgG) between seizure-free and seizure patients. Data are presented as individual values with mean ± SD. $^{*}p < 0.05$
