# Supplementary material for: Prognostic factors and a preliminary prognostic model in anti-GAD antibody-associated epilepsy
Source: Front Immunol. 2026 Feb 4;17:1738062. doi: 10.3389/fimmu.2026.1738062 (PMC12913182; doi:10.3389/fimmu.2026.1738062)
Supplement: Supplementary file 2 [file Image2.pdf]

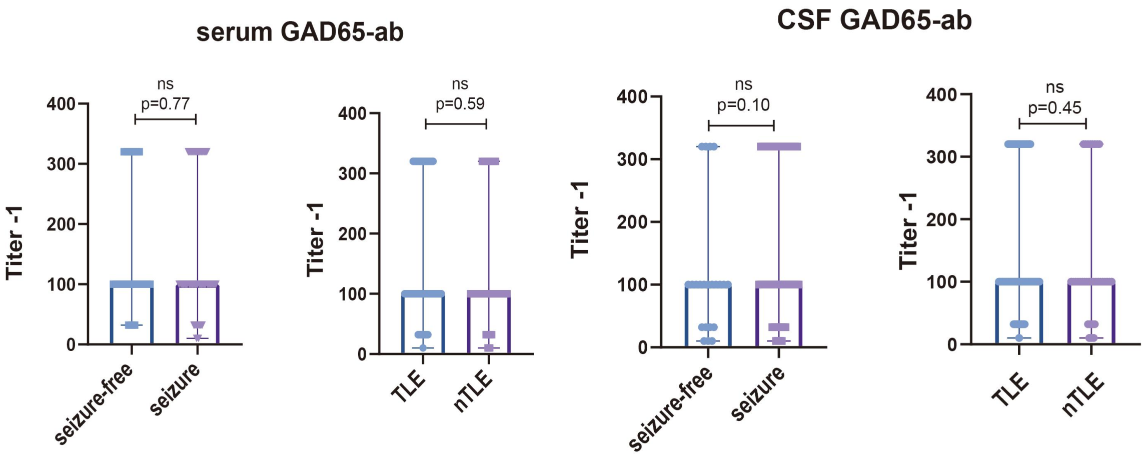

Figure S2. Association between anti-GAD65 antibody titers and prognosis and TLE

No significant association was observed between antibody titers and prognosis, nor between antibody titers and the presence of temporal lobe epilepsy (TLE). The x-axis represents patient groups, and the y-axis shows antibody titers^{-}1. Median and range are indicated.
