# Supplementary material for: Prognostic factors and a preliminary prognostic model in anti-GAD antibody-associated epilepsy
Source: Front Immunol. 2026 Feb 4;17:1738062. doi: 10.3389/fimmu.2026.1738062 (PMC12913182; doi:10.3389/fimmu.2026.1738062)
Supplement: Supplementary file 3 [file Table1.pdf]

Table S1. Distribution of outcomes in patients receiving monotherapy or combination first-line immunotherapy

	first-line immunotherapy alone	Combined first-line immunotherapy	total
seizure	28	37	65
seizure-free	13	8	21
Total	41	45	86

Note: Excludes 4 patients who did not receive immunotherapy and 1 patient whose immunotherapy regimen record was not detailed.
