# Supplementary material for: Prognostic factors and a preliminary prognostic model in anti-GAD antibody-associated epilepsy
Source: Front Immunol. 2026 Feb 4;17:1738062. doi: 10.3389/fimmu.2026.1738062 (PMC12913182; doi:10.3389/fimmu.2026.1738062)
Supplement: Supplementary file 4 [file Table2.pdf]

Table S2. Distribution of outcomes in patients treated with and without rituximab

	no RTX	RTX	total
seizure	47	19	66
seizure-free	17	4	21
Total	64	23	87

Note: Excludes 4 patients who did not receive immunotherapy. RTX rituximab
